# Rosuvastatin Decreases Intestinal Fatty Acid Binding Protein (I-FABP), but Does Not Alter Zonulin or Lipopolysaccharide Binding Protein (LBP) Levels, in HIV-Infected Subjects on Antiretroviral Therapy

**DOI:** 10.20411/pai.v1i1.124

**Published:** 2016-07-13

**Authors:** Nicholas T. Funderburg, Morgan Boucher, Abdus Sattar, Manjusha Kulkarni, Danielle Labbato, Bruce I. Kinley, Grace A. McComsey

**Affiliations:** 1 School of Health and Rehabilitation Sciences, Division of Medical Laboratory Science, Ohio State University, Columbus, Ohio; 2 Case Western Reserve University, Cleveland, Ohio; 3 University Hospitals Case Medical Center, Cleveland, Ohio; 4 Rainbow Babies and Children's Hospital, Cleveland, Ohio

**Keywords:** HIV-1, rosuvastatin, zonulin-1, intestinal fatty acid binding protein, lipopolysaccharide binding protein, inflammation

## Abstract

**Introduction:**

Altered gastrointestinal (GI) barrier integrity and subsequent microbial translocation may contribute to immune activation in HIV infection. We have reported that rosuvastatin improved several markers of immune activation in HIV+ participants, but the effect of statin treatment on markers of GI barrier dysfunction is unknown.

**Methods:**

SATURN-HIV is a randomized, double-blind, placebo-controlled trial assessing the effect of rosuvastatin (10mg/daily) on markers of cardiovascular disease, inflammation, and immune activation in ART-treated patients. Gut-barrier integrity was assessed by the surrogate markers intestinal fatty acid binding protein (I-FABP), a marker of enterocyte death, and zonulin-1, a marker of gut epithelial cell function. Levels of lipopolysaccharide binding protein (LBP) were measured as a marker of microbial translocation.

**Results:**

Rosuvastatin significantly reduced levels of I-FABP during the treatment period compared to the placebo. There was no effect of rosuvastatin treatment on levels of zonulin or LBP. Baseline levels of LBP were directly related to several markers of immune activation in samples from all participants, including soluble CD163, IP-10, VCAM-1, TNFR-II, and the proportion of CD4+ and CD8+ T cells expressing CD38 and HLA-DR. Many of these relationships, however, were not seen in the statin arm alone at baseline or over time, as inflammatory markers often decreased and LBP levels were unchanged.

**Conclusions:**

Forty-eight weeks of rosuvastatin treatment reduced levels of I-FABP, but did not affect levels of zonulin or LBP. The reduction in levels of inflammatory markers that we have reported with rosuvastatin treatment is likely mediated through other mechanisms not related to gut integrity or microbial translocation.

SATURN-HIV is registered on clinicaltrials.gov; Identifier: NCT01218802

**STANDFIRST**

Rosuvastatin treatment does not appear to improve GI-integrity in persons infected with HIV.

## INTRODUCTION

Immune activation and inflammation are hallmarks of chronic HIV infection, even when HIV+ individuals are receiving suppressive antiretroviral therapy (ART) [1, 2]. Several markers of immune activation, including interleukin-6 (IL-6), soluble CD14 (sCD14), and markers of T-cell activation, are associated with mortality in this population [3, 4]. Strategies to reduce immune activation in ART-treated HIV infection are being explored. Statins, or 3-hydroxy-3-methylglutaryl coenzyme A (HMG-CoA) reductase inhibitors, have anti-inflammatory effects [5, 6], and within the Stopping Atherosclerosis and Treating Unhealthy bone with RosuvastatiN in HIV (SATURN-HIV) trial, we have reported significant reductions in monocyte (sCD14 and tissue factor expression) and T-cell activation (CD38 and HLA-DR on CD4+ and CD8+ cells) and vascular inflammation (lipoprotein-associated phospholipase A 2 [Lp-PLA_2_]) following initiation of statin therapy [7–9]. The mechanism(s) related to statin-induced reduction of immune activation in HIV infection remain incompletely understood.

There are likely multiple contributors to immune activation in ART-treated HIV infection, including: low level HIV-1 replication [10]; copathogens [11]; pro-inflammatory lipids (e.g., oxidized LDL) [12, 13]; and microbial translocation [14]. Decreased GI integrity leads to increased circulating levels of lipopolysaccharide (LPS) in persons infected with HIV [15], and while ART often reduces plasma levels of LPS, these levels do not always normalize [16]. Plasma levels of LPS are directly related to T-cell and monocyte activation markers—including markers that predict mortality in HIV-infected individuals [17]—and LPS levels are related to coagulation markers in SIV-infected nonhuman primates [18]. Further, markers of gut epithelial barrier integrity (intestinal fatty acid binding protein [I-FABP] and zonulin-1) are also related to mortality in HIV-infected individuals [19]. We have reported previously that 48 weeks of statin therapy reduces cellular and plasma markers of monocyte, endothelial cell, and T-cell activation [7–9]. Here, we measured plasma levels of I-FABP, a marker of enterocyte death [20], zonulin-1, a marker of enterocyte function [21], and lipopolysaccharide binding protein (LBP), in order to determine whether the effect of statin therapy on reducing immune activation is mediated by improvement of GI integrity and reduction of microbial translocation.

## MATERIALS AND METHODS

### Study Design

Details of the trial design have been published [7–9]; in brief, SATURN-HIV is a randomized, double-blind, placebo-controlled study designed to measure the effect of rosuvastatin (10mg/ day) on markers of cardiovascular risk, skeletal health, and immune activation in HIV disease, and is registered on clinicaltrials.gov, Identifier: NCT01218802. SATURN-HIV was approved by the Institutional Review Board of University Hospitals Case Medical Center (Cleveland, OH) and all subjects signed a written consent prior to enrollment. The study was a 96 week trial that began with enrolling patients on 3/24/2011 and ended on 6/5/2014. Study drugs (active and placebo) were provided by AstraZeneca. All subjects were on stable ART for at least 3 months and cumulative ART duration of at least 6 months, with HIV-1 RNA <1,000 copies/mL, with fasting LDL-cholesterol (LDL-C) ≤130mg/dL and fasting triglycerides≤500mg/dL. Additional entry criteria included evidence of either heightened T-cell activation, identified as the proportion of CD8+ T cells that expressed CD38+HLA-DR+ ≥19%, or levels of high sensitivity C-reactive protein (hs-CRP) ≥2mg/L. Exclusion criteria included diabetes and known CVD.

### Blood/Sample Preparation

Whole blood samples were collected into EDTA-containing tubes. Plasma was isolated by centrifugation for 10 minutes at 400xg and was frozen at −80°C until thawed once and analyzed in batch. Plasma samples were collected at baseline, and at 24 and 48 weeks. Levels of zonulin-1 (ALPCO, Salem, NH), I-FABP (R&D Systems human FABP2 Duoset ELISA system: Minneapolis MN), and LBP (Hycult Biotech, Plymouth Meeting, PA) were measured by enzyme-linked immunosorbent assays (ELISA). Other plasma markers and cellular markers of immune activation were measured and have been described previously [8]. Levels of soluble CD14 and CD163 were measured using Quantikine enzyme-linked immunosorbant assay (ELISA) kits (R&D Systems, Minneapolis MN); levels of the proinflammatory cytokine IL-6 and soluble receptors of tumor necrosis factor α (sTNFR-I and sTNFR-II), interferon γ–inducible protein 10 (IP-10), the cellular adhesion molecules soluble vascular cell adhesion molecule 1 (sVCAM-1) and soluble intercellular adhesion molecule 1 (sICAM-1) were also measured by ELISAs (R&D Systems, Minneapolis, MN).

### T-Cell Phenotyping

The proportion of activated T cells required to determine eligibility was measured by analyses of freshly collected whole blood samples, processed as above. T cells were identified by size and granularity and by positive expression of CD3 and CD4 or CD8. T-cell activation was measured using anti-CD38 (PE), anti-HLA-DR (FITC), anti-CD3 (Peridinin Chlorophyll Protein Complex, PerCP), anti-CD8 (allophycocyanin-cy7, APC-cy7), and anti-CD4 (allophycocyanin, APC, all from BD Biosciences).

Assessment of T-cell activation for the entry, 24-, and 48-week timepoints was performed by comparing the expression of surface markers on cryopreserved PBMC samples from each patient. Samples were thawed and analyzed in batch. In addition to the T-cell markers described above, analysis of frozen PBMC samples also included a stain for cell viability (Live/Dead Violet, Pacific Blue) and an additional activation marker PD-1 (PE-Cy7, BD Pharmingen). Samples were analyzed using a Miltenyi MACS Quant flow cytometer (MiltenyiBiotec, BergischGladbach, Germany). MACS Quantify software (version 2.21031.1, MiltenyiBiotec) was used to analyze the data.

## STATISTICAL METHODS

Demographics, clinical characteristics, fasting metabolic parameters, and inflammatory and coagulation markers are described by study group. Continuous measures are described by medians and interquartile ranges, and nominal variables are described with frequencies and percentages.

Nominal variables were compared using χ^2^ analysis or Fisher's exact test. Continuous measures were tested for normality. For between-group comparisons (at baseline and changes from baseline to 48 weeks), normally-distributed variables were compared using t-tests, and non-normally-distributed variables were compared using Wilcoxon rank sum tests. For within-group changes from baseline to 48 weeks, normally-distributed variables were compared with a paired t-test, and non-normally-distributed variables were compared with Wilcoxon signed rank test. The non-parametric Spearman correlation was used to estimate correlations among changes in markers of inflammation and immune activation at week 48. Longitudinal changes in gut integrity bio-markers (IFAB, zonulin, and LBP) for rosuvastatin treatment were studied using the generalized estimating equation approach [22]. For visual comparison, the changes in gut biomarkers were also studied using graphs (box plot) over the 48-week period between the treatment groups.

All analyses were done using statistical software SAS 9.3 (SAS Institute Inc., Cary, NC, USA) and statistical software Stata 13.0 (College Station, TX).

## RESULTS

**Figure 1.**
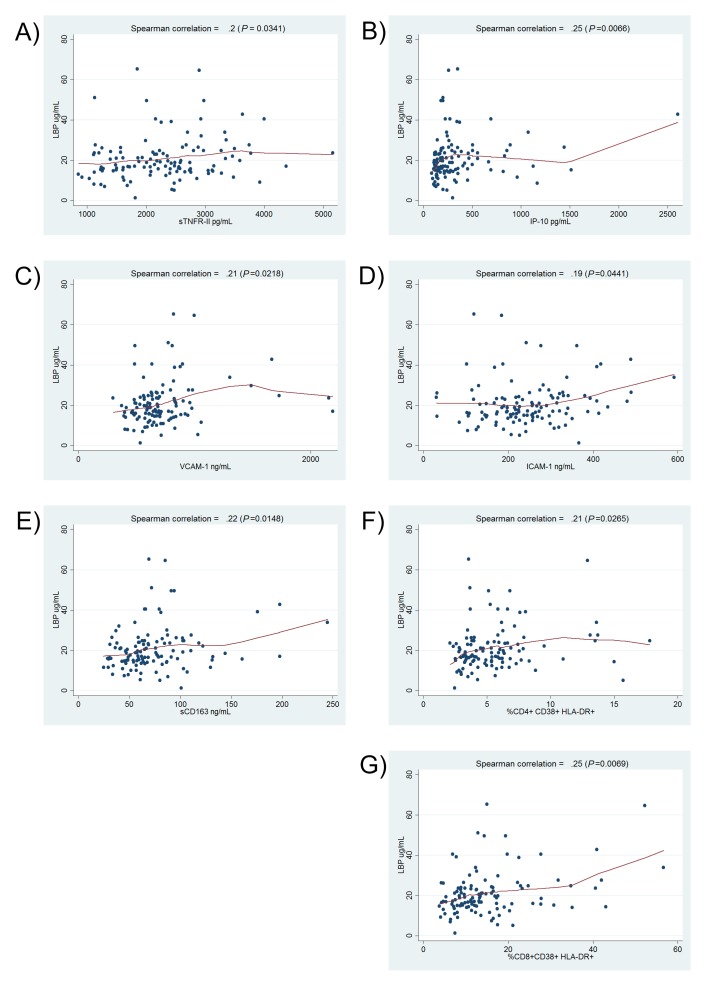
**Plasma levels of lipopolysaccharide binding protein (LBP) are related to markers of immune activation in HIV-infected patients.** Plasma samples were thawed and levels of (A) TNFr-II, (B) IP-10, (C) VCAM-1, (D) ICAM-1, and (E) sCD163 were measured by ELISA. Measurement of T-cell activation was performed by flow cytometry to assess surface activation markers (CD38, HLA-DR) on cryopreserved (F) CD4+ and (G) CD8+ T cells. Correlations between markers at baseline were determined using Spear-man rank correlation, and lines were fitted to the data points using locally weighted regression (LOWESS).

Baseline demographic and immunologic characteristics of study subjects have been published [7–9]; in brief, 147 participants were enrolled in the SATURN-HIV study (n=72 in the rosuvastatin arm; n=75 in the placebo arm). Overall, the median age of the participants was 47 years, 78% were male, 70% were African American, 29% were Caucasian, and 1% were Hispanic. There were no differences in markers of GI-integrity or microbial translocation at baseline between the study arms. Among all participants, baseline plasma levels of LBP were directly related to plasma levels of: interferon gamma induced protein 10 (IP-10, r= 0.25, *P*=0.007); tumor necrosis factor alpha receptor-II (TNFR-II, r=0.2, *P*=0.03); markers of monocyte activation (sCD163, r=0.22, *P*=0.015); endothelial cell activation (ICAM-1, r=0.19, *P*=0.04; VCAM-1, r=0.21, *P*=0.02); and the proportion of CD4+ (r=0.21, *P*=0.03) and CD8+ (r=0.25, *P*=0.007) T cells that expressed HLA-DR and CD38 (Figure 1). Among participants in the statin arm, only the relationships between LBP and sCD163 (r=0.3, *P*=0.019) and LBP and IP-10 (r=0.27, *P*=0.034) were significant. Baseline levels of I-FABP were inversely related to IP-10 levels (r=−0.18, *P*=0.05, not shown). Baseline levels of zonulin did not correlate with any of the markers we measured.

**Figure 2.**
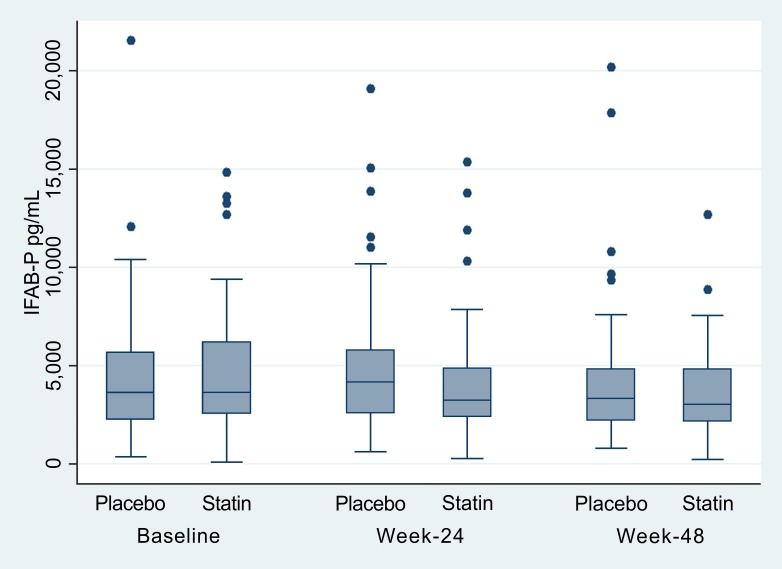
**Rosuvastatin treatment reduces levels of I-FABP, but not levels of zonulin or LBP.** Plasma samples were thawed and levels of intestinal fatty acid binding protein (I-FABP), zonulin, and lipopolysaccharide binding protein (LBP) were measured by ELISA. Rosuvastatin reduced levels of I-FABP over the study period compared to placebo. Levels of the other gut integrity markers did not change over time with statin treatment (not shown).

Rosuvastatin treatment was associated with reduced levels of I-FABP, when compared to placebo, during the 48-week treatment period (β=−1078.80 pg/mL, *P*=0.04, Figure 2). As I-FABP levels decreased overtime in the statin arm, we eventually measured an inverse relationship between sCD163 and I-FABP (r =−0.21, *P*=0.02) at week 48, but this relationship was driven by outliers within the data. Rosuvastatin treatment did not affect levels of LBP or zonulin, yet, a relationship between sCD163 and LBP was maintained at week 24, but became insignificant at week 48, likely due to the flat levels of LBP and a significant decrease in sCD163 at week 48 within the statin arm.

## DISCUSSION

The SATURN-HIV study is a double blind, randomized, placebo-controlled clinical trial of rosuvastatin as an immunomodulatory therapy in HIV-infected subjects on ART. We have reported that rosuvastatin decreases plasma and cellular markers of monocyte [8, 9] and T-cell activation [8], lowers vascular inflammation [7, 8], preserves renal function, and lowers cystatin C levels [23] in ART-treated HIV-infected persons. The direct mechanisms by which statin therapy improves chronic immune activation in these study participants are not fully understood.

Here we tested the hypothesis that rosuvastatin may modulate immune activation by improving the integrity of the GI tract and subsequently reducing microbial translocation. Bacterial products derived from the gut lumen, and specifically plasma levels of LPS, are directly related to T-cell activation (CD38 and HLA-DR expression) [15, 17], to levels of soluble CD14 [17], and to proportions of CD16+ monocytes [17], and are inversely related to CD4+ T cell reconstitution following initiation of ART [24]. Alternatively, if levels of inflammation and immune activation can be reduced with rosuvastatin treatment, the GI tract may recover normal barrier function. Therefore, by looking at the changes in inflammatory markers and gut barrier integrity markers serially over time, we may be able to infer causality.

Several markers of impaired GI-function have been measured in HIV-infected individuals [19, 25]. Plasma levels of I-FABP are increased in HIV-infected donors compared to uninfected donors [20] and increased plasma levels of I-FABP may represent increased enterocyte death, which may contribute to impaired barrier function. Zonulin-1 is produced by viable gut epithelial cells and plays a part in regulating paracellular intestinal permeability [21], and zonulin-1 has been found to be upregulated in different immune diseases that are characterized by chronic inflammation of the gut, including celiac disease [26]. Zonulin-1 and I-FABP levels are predictive of mortality in persons infected with HIV [19], so favorable modulation of their expression may improve clinical outcomes. We did not find statistically significant changes in zonulin-1 between the statin arm and the placebo arm. Levels of I-FABP decreased during statin treatment, which may represent decreased enterocyte death; perhaps 48 weeks of statin therapy is not long enough, or the local anti-inflammatory effect of rosuvastatin treatment was not potent enough to repair the GI tract sufficiently to measure changes in zonulin levels.

Levels of LBP, produced by hepatocytes in the liver, may reflect an attempt by the host to bind translocated LPS and clear it from the circulation [27]. Levels of LBP are increased in HIV-infected persons compared to uninfected controls [15] and LBP levels decrease following initiation of ART [28]. In our study, baseline levels of LBP were directly related to several markers of immune activation and inflammation (sCD163, CD38, and HLA-DR on T cells, among others) in all participants. Over 48 weeks of therapy, many inflammatory markers decreased with statin treatment [7–9], yet levels of LBP did not change significantly. Levels of sCD163 and LBP were directly related at week 24, but became insignificant at week 48, likely due to the flat levels of LBP and a significant decrease in sCD163 at week 48 within the statin arm. These baseline relationships did not persist over time, partially due to decreasing sample size (total population versus statin arm), but these results may also suggest that changes in microbial translocation and GI-function are not responsible for improvement in immune activation within these participants. This null result is made less surprising based on our recent findings that statin treatment reduces oxidized LDL (ox-LDL) levels in these subjects [29], and that early changes in oxLDL levels were related to changes in soluble CD14 and intima-medial thickness of the carotid artery. This suggests that statin-mediated decreases in pro-inflammatory lipid profiles may be on the causal pathway for reduction in inflammation, monocyte activation, and cardiovascular disease in ART-treated HIV infection.

While our results suggest that rosuvastatin treatment may not improve GI-tract permeability and result in decreased levels of microbial translocation, this current study has a few important limitations. First, we did not directly measure any microbial products (LPS, 16S rRNA, etc.) or their changes over time. These assays are technically complex and we chose not to perform them once we did not see significant changes in gut barrier markers. Second, binding and clearance of LPS from the circulation likely involves several mediators, including LBP, LPS binding antibodies (endoCab and secretory IgA), and lipid molecules [30]; measuring LBP alone likely does not provide a complete picture of the mechanisms of LPS clearance. We also did not directly measure endothelial cell/tissue structure, expression of gut barrier proteins, or the transcriptional profile of endothelial cells, which has been shown to be important in measuring GI permeability in HIV infection [31]. Further tests that directly measure barrier protein expression and/or GI permeability by lactulose/mannitol absorption [32] would be required to definitively assess GI barrier function; these assays are not possible in the current study.

Overall, our findings suggest that decreases in inflammation and immune activation in ART-treated HIV-infected persons following rosuvastatin treatment are not likely attributable to decreases in microbial translocation and improvements in GI-barrier function.
